# Clinical Insights Into Ductal Carcinoma In Situ in Males: A Report of Two Cases From the Sultanate of Oman

**DOI:** 10.7759/cureus.71071

**Published:** 2024-10-08

**Authors:** Mohamed J Eshaqi, Salma A Al Shamsi, Amani M Al Saidi, Smitha Mahesh

**Affiliations:** 1 Department of General Surgery, Armed Forces Hospital, Muscat, OMN

**Keywords:** breast and endocrine surgery, breast cancer pathology, breast conservative surgery, dcis, ductal carcinoma in situ, male breast cancer, male breast carcinoma, malignancy of breast in male, plastic and reconstructive surgery, stigmas of male breast cancer

## Abstract

Breast cancer is a disease that predominantly affects the female population; however, rarely it can manifest in males, yet its etiology remains poorly elucidated. The scarcity of literature reviews and case reports done on male breast cancer in comparison to the female counterpart makes it difficult to understand the risk factors, treatment options, and extension of the disease. Moreover, high-grade ductal carcinoma in situ (DCIS) is exceptionally uncommon among male patients. The prognosis for male patients diagnosed with DCIS is similar to that of females at the same disease stage making early recognition and diagnosis significant. However, more efforts are being made to understand the clinical presentation, increase awareness, and acknowledge the etiology of this disease. This study addresses this gap by presenting two distinctive cases of invasive ductal carcinoma in males from the Sultanate of Oman. The aim of this study is to contribute to the growing efforts in comprehending the unclear landscape of male breast cancer, fostering awareness, and advancing knowledge of its etiology.

## Introduction

Male breast cancer is a rare disease that accounts for approximately 1% of all breast cancer cases [1]. The incidence of breast cancer in males is reportedly increasing, highlighting an emerging trend [2]. However, the specific factors influencing this increase and the features of its clinical presentation in men remain largely unknown. Ductal carcinoma in situ (DCIS) in males is exceedingly rare and the literature reported is very limited. Shedding light on the early detection of DCIS can have a significant impact on mortality, if recognized. While DCIS is rare, mortality rates in male cases are 19% higher than in their female counterparts which raises the importance of surveillance and tailoring treatment to the patients [3]. The mean age of onset for male breast cancer typically falls within the range of 60 to 69 years old; there is a concerning trend as cases continue to be reported among younger men, emphasizing the importance of broadening our understanding of the disease across diverse age groups [4]. We present two cases of young men who have been diagnosed with invasive ductal carcinoma and the treatment they have undergone.

## Case presentation

Case 1

A 47-year-old male presented to the clinic with a chief complaint of a breast lump that had been progressively increasing in size over the past three months. The patient reported no associated pain, nipple discharge, or other breast-related symptoms. Notably, he had no significant past medical history, no family history of breast cancer, and no history of smoking or alcohol consumption. He is married and has children.

Upon physical examination, the patient was thin built and afebrile and had no apparent signs of pallor. Breast examination revealed a well-defined 20mm cystic lump located under the left nipple areolar complex. The lump exhibited mobility and was not fixed to the skin or underlying pectoralis major muscle or fascia. Ulcerations, peau d’orange, and skin pigmentations were absent and the nipple areolar complex appeared normal. Importantly, there were no palpable axillary nodes, and examination of the right breast revealed no similar lump. 

**Figure 1:**
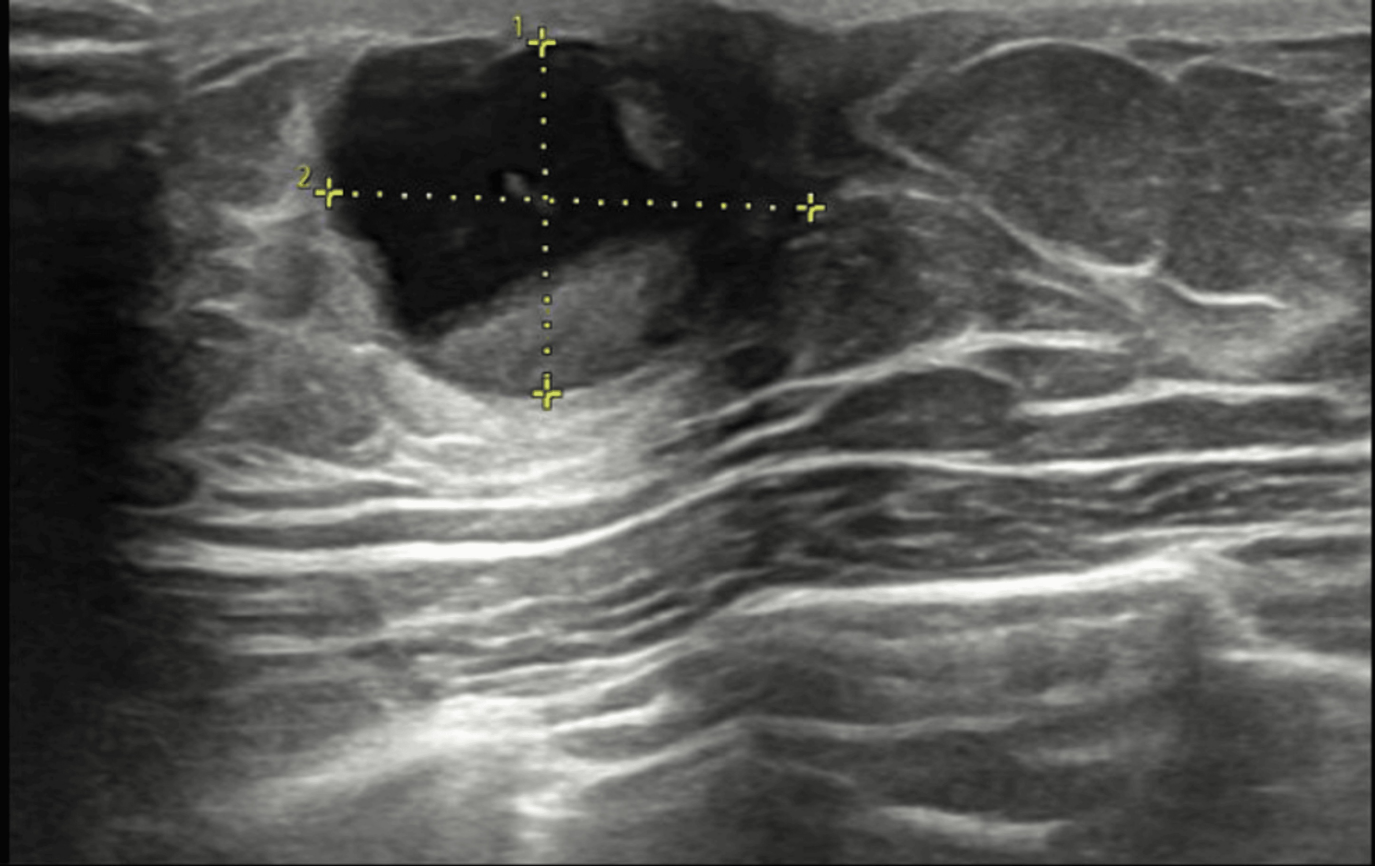
Ultrasonography breast done as a part of the triple assessment The well-defined oval-shaped mixed solid and cystic lesion measured 15 x 13 mm and was located at the 12 o'clock position of the left breast. The lesion shows a solid component adherent to the wall measuring approximately 10x11mm. Imaging features were consistent with a suspicious breast lesion. No other lesions in the left breast were seen. Suspicious-appearing lymph nodes were noted in the left axilla with maintained fatty hilum.

An initial diagnostic step was ultrasonography, which revealed a mixed 15x13 mm solid and cystic lesion at the 12 o'clock position of the left breast (Figure 1). Subsequently, fine needle biopsy was performed, and the cytology report categorized the lesion as C3 - an atypical lesion, as per the International Academy of Cytology (IAC) system for breast cytology reporting.

Further investigations involved a breast lump excision, and the subsequent histopathology report unveiled an encysted papillary carcinoma, specifically of the papillary type DCIS, measuring 10mm. Additionally, foci of ductal carcinoma in situ were identified, displaying intermediate nuclear grade, with cribriform and solid types present. Immunohistochemical studies demonstrated a strongly positive profile for estrogen receptors, progesterone receptors, and HER-2.

**Figure 2:**
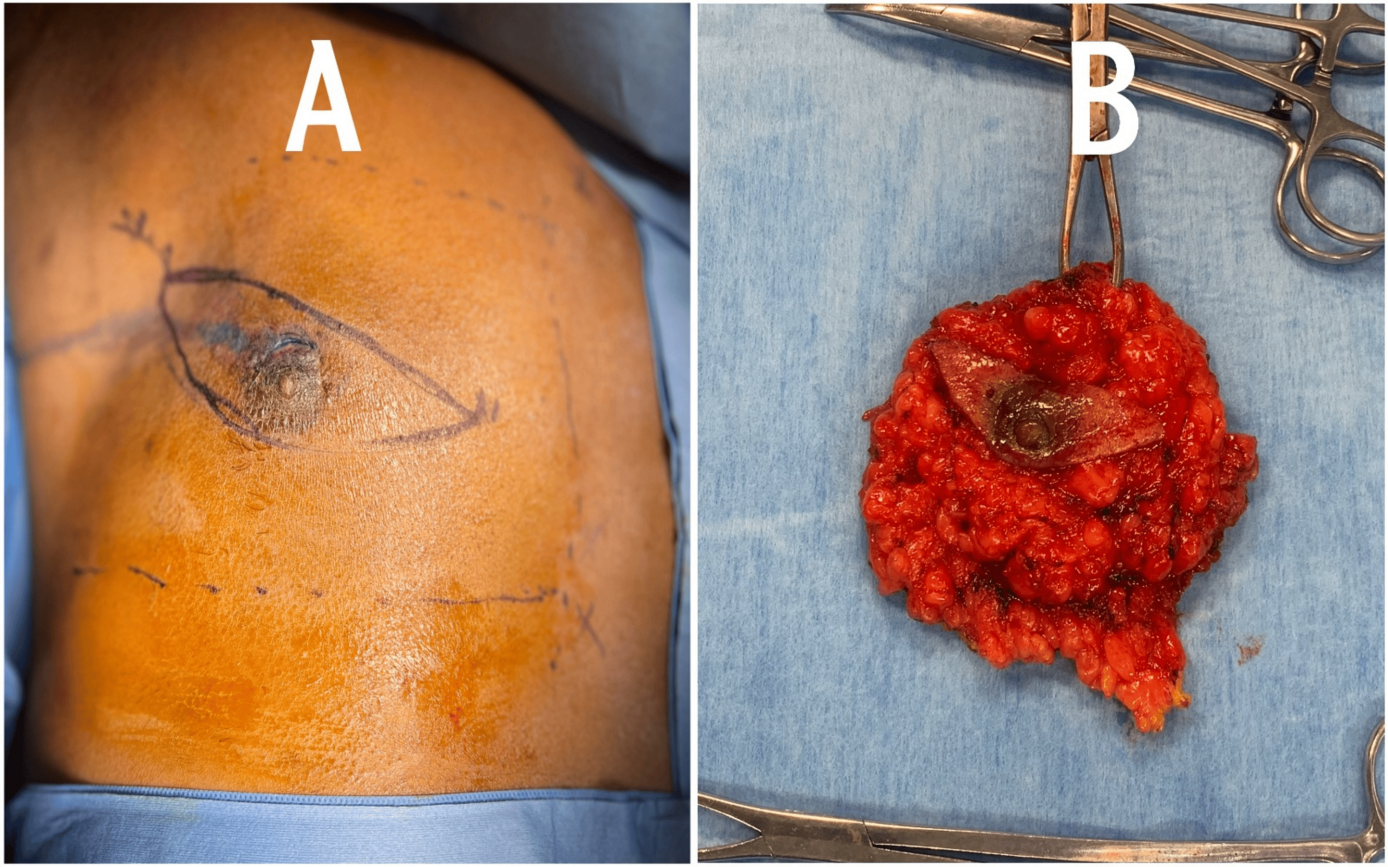
Pre-operative and post-operative demonstrations (A) The enlarged left breast with pre-operative elliptical markings indicating the planned surgical incisions. (B) The excised breast tissue specimen following the left breast mastectomy, providing a clear view of the tissue removed during the procedure.

In light of the histopathological findings, a comprehensive discussion was conducted with the patient to explore suitable management options. After thorough consideration of the available choices, the patient opted for a left breast mastectomy as the chosen course of action. This decision was made collaboratively, taking into account the specific characteristics of the lesion and the patient's individual circumstances. As seen in Figure 2A, pre-operative markings on the left breast indicate an elliptical type of incision, while Figure 2B shows the excised breast tissue post-operatively following the mastectomy.

Case 2

A 41-year-old male presented with complaints of right breast enlargement persisting for the past year, accompanied by intermittent pain and nipple discharge. His medical history revealed a decade-long use of hormonal medications for bodybuilding and athletic purposes. Despite controlled hypertension and smoking, he had no recorded medications or diabetes.

On examination, he appeared afebrile with normal secondary sexual characteristics and no pallor. Breast examination revealed a well-prominent right nipple-areolar complex with a firm, mildly tender, large palpable lump measuring approximately 30x40mm. There were no active nipple discharges or skin changes noted. Bilateral axillary lymph nodes were palpable but non-tender, with unremarkable findings on abdominal and scrotal examination.

**Figure 3:**
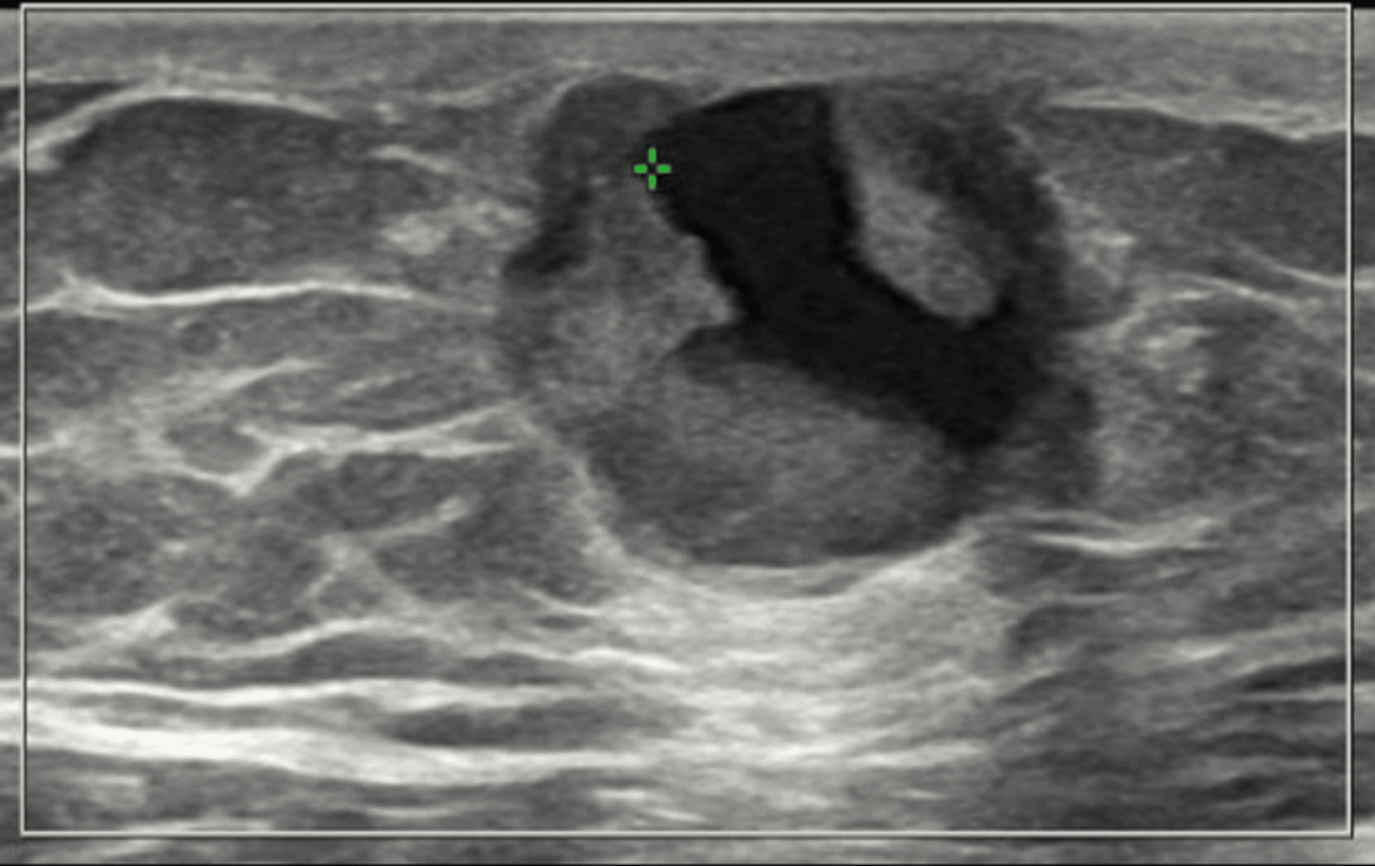
Right breast ultrasonography showing a solid-cystic mass in the right retroareolar region The lobulated solid-cystic hypoechoic mass measured 15 x 27 x 25 mm, located in the right retroareolar area. Doppler imaging revealed vascularity within the mass. Imaging features were consistent with a primary malignant breast lesion. No other lesions in the right breast were seen. Suspicious-appearing lymph nodes were observed in the right axilla.

Figure 3 illustrates the ultrasonography findings, which revealed a lobulated solid-cystic hypoechoic mass in the right retroareolar area, displaying vascularity on Doppler. The mass measures 15x27x25 mm, and there are suspicious lymph nodes present. Fine needle biopsy confirmed a proliferative breast lesion with mild atypia, leading to a diagnosis of invasive DICS, high grade, with ER and PR positivity.

Considering the findings, the patient underwent simple mastectomy following counseling, aiming at curative management.

## Discussion

The incidence of DCIS in the male breast is extremely rare. There are very few cases of pure DCIS in the literature to date. Generally, male breast cancer presents as an invasive carcinoma and the most common dominant subtypes of DCIS in males are papillary and cribriform respectively, and it has been shown that DCIS is less likely to be of the comedo subtype [5]. Compared to its female counterpart, DCIS in males tends to present at a more advanced stage, and at a later age it tends to present as a retroareolar, palpable mass, and in many cases bloody nipple discharge [5]. While the majority of the studies revolve around screening, workup, and management of breast cancer in females, understanding the unique aspects of male breast cancer is crucial for improved diagnosis, treatment, and outcomes. Similarly to female breast cancer assessment, the triple assessment, involving clinical evaluation, histological examination, and imaging studies, forms the cornerstone of male breast carcinoma initial evaluation. Several imaging studies may aid in clinical diagnosis such as ultrasonography, mammography, and MRI; however, in our subjects, the only imaging study done was ultrasonography due to the availability at the institution.

Current guidelines for male breast cancer treatment, as outlined by organizations like the American Society of Clinical Oncology (ASCO), emphasize a multimodal approach similar to that for female breast cancer. Surgery remains the cornerstone, with options ranging from mastectomy to lumpectomy, often followed by radiation therapy. Hormone receptor-positive tumors are typically managed with hormonal therapies such as tamoxifen or aromatase inhibitors. Chemotherapy and targeted therapies like trastuzumab are employed based on tumor characteristics, while genetic testing may guide decisions regarding inherited mutations. Regular follow-up is crucial to monitor for recurrence. These guidelines aim to optimize outcomes while addressing the unique aspects of male breast cancer biology [6]. Breast-conserving surgery is also an option opted for by most patients due to cosmetic considerations, but it is worth keeping in mind that this technique is hugely influenced by the mass of breast tissue concentrated under the nipple, which is usually a limitation for male patients as they have limited breast tissue. 

Genetic predispositions play a significant role in male ductal carcinoma, with a notable portion of cases reporting familial history, underscoring the importance of genetic screening. Mutations in genes such as BRCA2 and PALB2 have been identified as contributors, with some cases associated with Klinefelter syndrome, emphasizing the diverse genetic landscape [7]. 

In discussing the presented case of a male patient who developed breast cancer following long-term hormonal therapy for bodybuilding, it is important to consider the existing scientific literature on this potential correlation. Anabolic steroids, commonly used by athletes to enhance muscle mass and performance, can significantly alter hormonal balances, notably increasing estrogen levels as a result of the aromatization of excess testosterone. This change in hormonal milieu may contribute to the development of breast tissue abnormalities, including estrogen-dependent male breast cancer [8]. While there is biological plausibility in the connection between anabolic steroids and male breast cancer, scientific research specifically addressing this link remains limited. Most available studies focus on the broader endocrine effects of anabolic steroids, with fewer directly examining their impact on male breast cancer incidence. This indicates a need for more targeted research to better understand the risks and mechanisms involved, highlighting the importance of clinical vigilance and awareness regarding the long-term use of performance-enhancing drugs.

Environmental factors also warrant consideration in the discussion of male ductal carcinoma. Occupational exposures to certain chemicals and electromagnetic fields have been implicated, presenting potential avenues for preventive measures. Additionally, recent epidemiological findings highlight the role of moderate alcohol consumption as a risk factor, contributing to the multifaceted etiology of male breast cancer [9].

Advancements in genomic profiling have paved the way for precision medicine in male ductal carcinoma. Tailored treatment approaches, guided by the unique genetic makeup of the tumor, enhance therapeutic efficacy and mitigate adverse effects. Furthermore, emerging studies exploring the potential benefits of immune checkpoint inhibitors in male breast cancer cases underscore the need for continued research in this domain.

Furthermore, shedding light on the social stigma associated with male breast cancer profoundly affects those diagnosed with the disease. Its rarity means it receives minimal media coverage and public attention, reinforcing the misconception that breast cancer is solely a women's issue. This lack of visibility leads to feelings of invisibility and isolation for men with the condition. Friends, family, and coworkers often struggle to understand or empathize with their experiences, which heightens their sense of alienation. Additionally, societal expectations about masculinity can prevent men from discussing their diagnosis openly, as they may fear judgment or ridicule. This reluctance to share their experiences hinders awareness and support, perpetuating the stigma and misunderstanding surrounding male breast cancer.

Despite progress, challenges persist in male ductal carcinoma research. Limited epidemiological studies and a smaller pool of clinical data necessitate larger-scale collaborative efforts to comprehensively understand the disease's nuances. Furthermore, the rarity of male breast cancer emphasizes the importance of multi-center studies to accumulate a robust dataset for evidence-based decision-making.

## Conclusions

In conclusion, male DCIS presents a unique challenge in the realm of breast cancer, requiring an extensive understanding of its genetic, hormonal, and environmental factors. Collaborative research endeavors, informed by the evolving landscape of precision medicine, hold the key to refining diagnostic approaches and tailoring effective therapeutic strategies for optimal outcomes in male breast cancer cases. Finally, there is no difference in prognosis between male and female breast carcinoma when compared at similar stages, and thus, it is crucial to recognize male breast carcinoma in its earlier stages. In both genders, DCIS if diagnosed at an early stage has a favorable prognosis.
